# Role of the Exogenous HCV Core Protein in the Interaction of Human Hepatocyte Proliferation and Macrophage Sub-Populations

**DOI:** 10.1371/journal.pone.0108278

**Published:** 2014-09-29

**Authors:** Zhiyan Yao, Xiaotian Song, Shiru Cao, Wenzhang Liang, Wenran Lu, Lijuan Yang, Zhengzheng Zhang, Lin Wei

**Affiliations:** 1 Department of Immunology, Hebei Medical University, Shijiazhuang, China; 2 Key Laboratory of Immune mechanism and Intervention on Serious Disease in Hebei Province, Shijiazhuang, China; Saint Louis University, United States of America

## Abstract

**Background:**

The core protein of hepatitis C virus (HCV) is found in the cytoplasm and nuclei of infected cells, including hepatocytes and other cells in the liver. The core protein could be secreted as well. Resident liver macrophages are dependent on the tissue micro-environment and external stimuli to differentiate M1 and M2 hypotypes with distinct functions, and increased expression of the nuclear transcription factor STAT3 was seen in M2-polarized macrophages. In contrast to proinflammatory M1 macrophages, M2 macrophages serve beneficial roles in chronic inflammation, immunosuppression, and tumorigenesis.

**Methods:**

Monocyte-derived human macrophage line (mTHP-1) was treated with the exogenous HCV core protein. Next, the mTHP-1 culture supernatant or cell pellets were added to culture media of normal human liver cell line (L02).

**Results:**

Only the culture supernatant stimulated L02 cells proliferation, which was associated with phosphorylated ERK expression. Core protein activated mTHP-1 cells showed enhanced pro- and anti-inflammatory cytokines secretion, which was accompanied by high expression of phosphorylated NF-κB105 and NF-κB65. However, phosphorylated STAT1, and STAT3, which are normally associated with M1 and M2 macrophage polarization, and cell surface expression of CD206, CD14, CD16, and CD86, were unaltered. A transwell co-culture system showed that only in mTHP-1 co-cultured with L02 in the presence of exogenous core protein, were higher levels of phosphorylated STAT3 and CD206 seen.

**Conclusions:**

We showed L02 cells proliferation was accelerated by the culture supernatant of mTHP-1 cells treated with the exogenous HCV core protein. The exogenous core protein mediated the interaction between macrophages and hepatocytes in co-culture, which enhanced the expression of phosphorylated STAT3 and CD206 in macrophages.

## Introduction

Hepatitis C virus (HCV) is a hepatotropic virus, which upon infection, is a major cause of liver fibrosis, cirrhosis, and hepatocellular carcinoma (HCC) [1], [2]. HCV not only infects hepatocytes, also replicates in many immune cells (e.g., B cells, T cells, monocytes and macrophages) to affect their functions [3], [4]. During the long developing period from HCV infection to HCC, immune cells are recruited to the liver in an attempt to control viral replication; however, in majority of cases chronic infection is established. In addition, HCV and viral proteins could activate hepatocytes, immune cells (e.g., macrophage, dendritic cells, and natural killer cells), and stromal cells (e.g., stellate cells, and myofibroblasts) to escape host immune defense in the inflammatory micro-environment [5]–[7], which might contribute to the development of disease. However, the molecular mechanisms by which HCV and its proteins modulate host immunity and contribute to disease is poorly understood.

The HCV core protein is an RNA-binding protein, which participates in the formation of the viral nucleocapsid and modulates a series of biochemical process of host cells. These processes include cell growth, metabolism, apoptosis, carcinogenesis, and immune modulation [8]–[10]. It has been reported that the proliferation of HepG2 cells expressing the HCV core protein was provoked through promoting autocrine secretion of a heparin-binding EGF-like growth factor (HB-EGF), and activating Akt by the Ras/PI3K signaling pathway [11]. Additionally, Benzoubir have revealed that hepatocyte Huh7 cells expressing the HCV core protein could secrete TGF-β and activate stellate cells in co-culture [12]. Kupffer cells (KCs), resident liver macrophages, play an important role in immune surveillance and immunoregulation. Different subtype macrophages are involved in the inflammatory micro-environment, including classically activated macrophages (M1 macrophages), which mediate host defense and antitumor immunity; alternatively activated macrophages (M2 macrophages), which suppress inflammatory responses and promote wound healing; tumor-associated macrophages (TAM), which suppress tumor immunity; the monocytic subset of myeloid-derived suppressor cells (MDSCs, which are functionally similar to TAMs) and regulatory macrophages, which predominantly secrete IL-10, and many other different cytokines, play differential roles in viral infection and tumor formation. Although there are some differences among the M2, TAM, MDSC and regulatory subsets of macrophages, each of these populations has a predominant immunosuppressive activity [13]. However, under the liver micro-environment, the role of HCV core protein interaction with macrophages remains largely unclear. Such as, how it induces the macrophage subtypes switch and its specific influence during the different subtype macrophages interaction with hepatocytes.

In the current study, the supernatant obtained from macrophages treated with the exogenous core protein stimulated proliferation of human hepatocytes. Additionally, the exogenous core protein did not only increase the expression of IL-1β, IL-8, TNF-α, TGF-β, IL-12 at mRNA level in macrophages but also induce the TGF-β secretion. This was accompanied by high expression of phosphorylated NF-κB105 and NF-κB65. However, there was no effect on the expression of membrane-associated CD206, CD14, CD16, and CD86, or the transcription factors (STAT1 and STAT3) by macrophages. Using a transwell co-culture system, we were surprised that the exogenous core protein increased phosphorylated STAT3 and CD206 in macrophages and phosphorylated ERK in hepatocytes on co-culturing these cells. It suggests that the exogenous core protein stimulates hepatocytes via specific pathway to induce a distinct set of molecules, and this culminates in the generation of altered macrophage subtype. Although the specific mechanisms remain to be confirmed, we have described novel insights into the role of a viral protein in the liver micro-environment, where it mediates novel interaction between hepatocytes and macrophages.

## Materials and Methods

### Cell culture and the expression of HCV core protein

The acute monocytic leukemia cell line THP-1 (obtained from the resource center of Peking Union Medical College Hospital, China), and the normal human liver cell line L02 (purchased from shanghai fuxiang Biotechnology Co., Ltd., China), were cultured in RPMI 1640 (Gibco) that was supplemented with 10% (vol/vol) fetal bovine serum (HyClone), penicillin-streptomycin (100 µg/ml for each drug, Solarbio), at 37°C with 5% CO_2_ in a humidified atmosphere. Before each test, monocytes (THP-1) differentiate to macrophages (mTHP-1) after being induced by phorbol 12-myristate 13-acetate (PMA, 40 ng/ml, ENZO) for 48 h.

HCV core gene fragment was obtained from the plasmid containing the full gene JFH-1 strain (a gift from Professor Yue Wang, Institute of Virus research, Center of Disease Control and Prevention, China). A recombinant prokaryotic expression plasmid pET28a/HCV core and eukaryotic expression plasmid pEGFP-C1/HCV core was constructed. Next, the recombinant core protein with six histidine residues fused at the N terminus was expressed and purified under native conditions in our laboratory. The HCV core protein, which was >95% pure, as determined by 10% SDS-PAGE, and specific hybridization with the mouse anti-human HCV core monoclonal antibody (sc-57800, Santa Cruz) were observed by Western blot. Limulus reagents were used to analyze the content of contaminating endotoxin in prokaryotic expressed proteins. To verify the biological activity of the recombinant protein, the mRNA expression level of a complement receptor C1qR of mTHP-1 cells treated with different concentrations the HCV core protein was detected by real time PCR. The mTHP-1 cells that were treated with PBS or LPS (Sigma, USA) served as the controls.

The eukaryotic expression plasmid pEGFP-C1/HCV core was transfected into L02 cells by Liposome 2000 reagent (Invitrogen, USA), and plasmid pEGFP-C1, which was transfected into L02 cells as the control. Subsequently, the L02/pEGFP-C1-core and the L02/pEGFP-C1 expressing cells were respectively screened by G418 (Invitrogen, USA) selection and then identified by Western blot analysis. Glutathione S-transferase (GST), and the M protein of group A streptococcus (GAS/M) proteins, which were used as the control proteins in the study, were purified as previously described [14], and then boiled for 30 min to form inactive-GST and inactive-GAS/M proteins.

### Liver cell proliferation analysis

For liver cell proliferation analysis, the following analyses were performed. 1) Preparation of mTHP-1 cells culture supernatant, and mTHP-1 cell pellets. For this analysis, culture supernatant was obtained from mTHP-1 cells that were treated with HCV core protein for 24 h. Additionally, the mTHP-1 cell pellets were obtained after treatment with the HCV core protein for 24 h following additional treatment with mitomycin C (10 µg/ml, Solarbio) for 1 h. 2) L02 cells were cultured in different ratios of the diluted cell culture supernatant in standard cell culture medium (i.e., 10% supernatant and 90% complete medium; 20% supernatant and 80% complete medium; 40% supernatant and 60% complete medium). Alternatively, L02 cells were co-cultured with the collected mTHP-1 cell pellets, where in the ratio of liver cells and mTHP-1cells were 25∶1 or 50∶1. 3) Dynamic changes of L02 cells proliferation were analyzed with the Cell Counting Kit-8 (CCK8, Dojindo) at different time points (i.e., 24 h, 48 h, and 72 h). The control groups were L02 cells that were treated with cell culture supernatant of PBS exposed mTHP-1 cells or complete medium that contained the core protein alone. We also assayed L02 cells that were co-cultured with mTHP-1 cell pellets treated with PBS as the control group.

### Real time PCR

The mRNA expression of C1qR and TLR4 of mTHP-1 cells treated with the different concentrations HCV core protein was detected. And after being treated with the HCV core protein of optimized concentration (10 µg/ml), the mRNA expression of the pro-inflammatory and anti-inflammatory cytokines (i.e., IL-1β, TNF-α, IL-12, IL-8, and TGF-β) of mTHP-1 cells was detected. The primer sequence pairs were designed using primer5 and NCBI online primer blast software.

The primer sequence pairs for C1qR were sense: 5′-GACACGCCTTACTCTAACTGG-3′ and antisense: 5′-AGCCCTCAATGTTACTTCCG-3′.

The primer sequence pairs for TLR4 were sense: 5′-TGAAACCCAGAGCTTTCAGACTCC-3′ and antisense: 5′-GGAGGTTGTCGGGGATTTTGTAG-3′.

The primer sequence pairs for IL-1β were sense: 5′-TGATGGCTTATTACAGTGGC-3′ and antisense: 5′-TGTAGTGGTGGTCGGAGATT-3′.

The primer sequence pairs for TNF-α were sense: 5′-CCTGGTATGAGCCCATCTAT-3′, and antisense: 5′-ACAGGGCAATGATCCCAAAGTA-3′.

The primer sequence pairs for IL-12 were sense: 5′-TCTTCATCAGGGACATCATCAA-3′, and antisense: 5′-CAGGGAGAAGTAGGAATGTGGA-3′.

The primer sequence pairs for IL-8 were sense: 5′-ACTTAGATGTCAGTGCATAAAGAC-3′, and antisense: 5′-TTATGAATTCTCAGCCCTCTTCAA-3′.

The primer sequence pairs for TGF-β were sense: 5′-TCCTGGCGATACCTCAGCAACC-3′, and antisense: 5′-CGCTAAGGCGAAAGCCCTCAAT-3′.

The primer sequence pairs for GAPDH were sense: 5′-CCTCTGACTTCAACAGCGACAC-3′, and antisense: 5′-CACCACCCTGTTGCTGTAGCCA-3′.

The PCR protocol was as follows: pre-denature at 95°C for 30 s for 1 cycle, denature at 95°C for 5 s, and anneal at 60°C for 30 s for 35 cycles using an enzyme dye mixture from the SYBR Green PCR Kit (TaKaRa).

### Flow cytometry analysis

The mTHP-1 cell suspension of the experimental group or control groups was adjusted to 10^7^ cells/ml with RPMI 1640 culture medium. Next, 100 µl of the suspended cells were incubated for 25 min at room temperature in a dark area with 8 µl of each of the mouse anti-human CD14, CD16, CD206 or CD86 monoclonal antibodies (Becton, Dickinson and Company, USA) that were respectively conjugated to PerCP, FITC, APC, and PE. The mouse IgG1 isotypes were used as controls. Suspensions were washed twice with PBS buffer and analyzed by flow cytometry (Becton, Dickinson and Company, USA). Data were analyzed by CellQuest software. The mTHP-1 cells that were treated with PBS or LPS or L02 cells (by transwell co-culture system) alone were used as the control groups for this analysis.

### Enzyme Linked Immunosorbent Assay (ELISA)

ELISA was used to detect TGF-β level in cell-free culture supernatant of mTHP-1 cells that were treated with the HCV core protein (10 µg/ml) for 24 h according to the manufacturer’s instructions (NeoBioscience Technology Co., Ltd., china). The cell-free culture supernatant of mTHP-1 cells that were treated with PBS or LPS alone served as the control groups.

### Western blot analysis

Protein concentrations in the cell lysates were determined by Nanodrop ND-1000 (Gene Co., Ltd., Hong Kong, china). Once protein concentrations were determined, 50 µl of the protein specimens were denatured in 50 µl of 2×sample buffer (125 mmol/L Tris-HCl, pH 6.8, 20% glycerol, 10% β-mercaptoethanol, 0.02% bromophenol blue, and 4% SDS). Briefly, 30 µg of denatured protein were separated using a sodium dodecyl sulfate polyacrylamide gel electrophoresis (SDS-PAGE) and transferred to a polyvinyl difluoride (PVDF) membrane, which was blocked in 5% milk proteins that were suspended in tris-buffered saline tween-20 (TBST) for 2 h at room temperature. The membrane was rinsed three times with TBST followed by incubation with the primary antibody (Cell Signaling Technology, Inc, USA) overnight at 4°C. The membrane was then washed and treated with anti-rabbit secondary antibodies that were conjugated with horseradish peroxidase at a 1∶5000 dilution (Bioworld Technology Inc, USA). The immunoreactive proteins were visualized with a chemiluminescence detection kit (PerkinElmer, USA), and the expression of glyceraldehyde-3-phosphate dehydrogenase (GAPDH) protein was used as the loading control across all samples analyzed by Western blot.

### Statistical Analysis

The experiments described in this study, were performed in triplicate. Results are expressed as mean ± one standard deviation about the mean. Statistical analyses for multiple comparisons were conducted using the one-way analysis of variance (ANOVA) function. All analyses mentioned above were fulfilled using the SPSS version 16.0 software program. Alpha values of *p*<0.05 were considered to indicate a statistically significant difference between compared data.

## Results

### The biological activity of the recombinant HCV core protein

**Figure 1:**
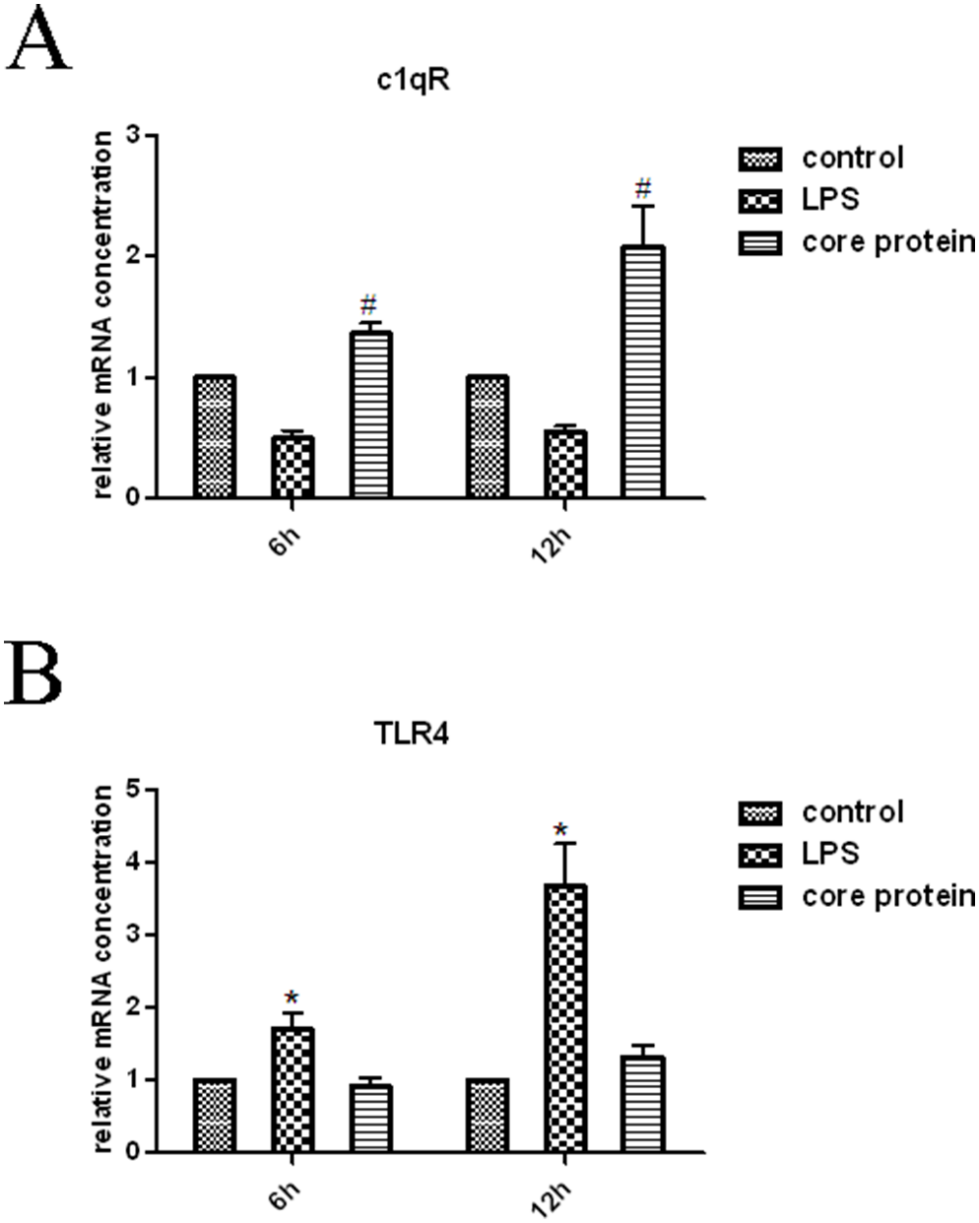
The biological activity of the recombinant HCV core protein. Before each test, the monocytes (THP-1) differentiate to macrophages (mTHP-1) after incubation with PMA (40 ng/ml) for 48 h. Real-time PCR analysis of C1qR and TLR4 that expressed by mTHP-1 cells treated with PBS (control) or LPS (1 µg/ml) or HCV core (10 µg/ml). (A) The mRNA level of C1qR. #*p*<0.05 indicates alpha value as compared to control and LPS groups; (B) The mRNA level of TLR4. *p<0.05 indicates alpha value as compared to control and HCV core protein groups.

To verify the biological activity of the recombinant HCV core protein, the mRNA expression level of the C1qR of mTHP-1 cells treated with the different concentrations core protein was detected by RT-PCR. It showed that C1qR, a receptor of HCV core protein [15], was significantly increased in experimental group (mTHP-1 treated with 10 µg/ml core protein) as compared to the PBS and LPS control groups (Figures 1A). However, the TLR4, a receptor of LPS, was elevated in the LPS control group (Figures 1B). The result is in accordance with the previous report [16].

### Supernatant from mTHP-1 treated with the exogenous HCV core protein stimulated liver cell proliferation

**Figure 2:**
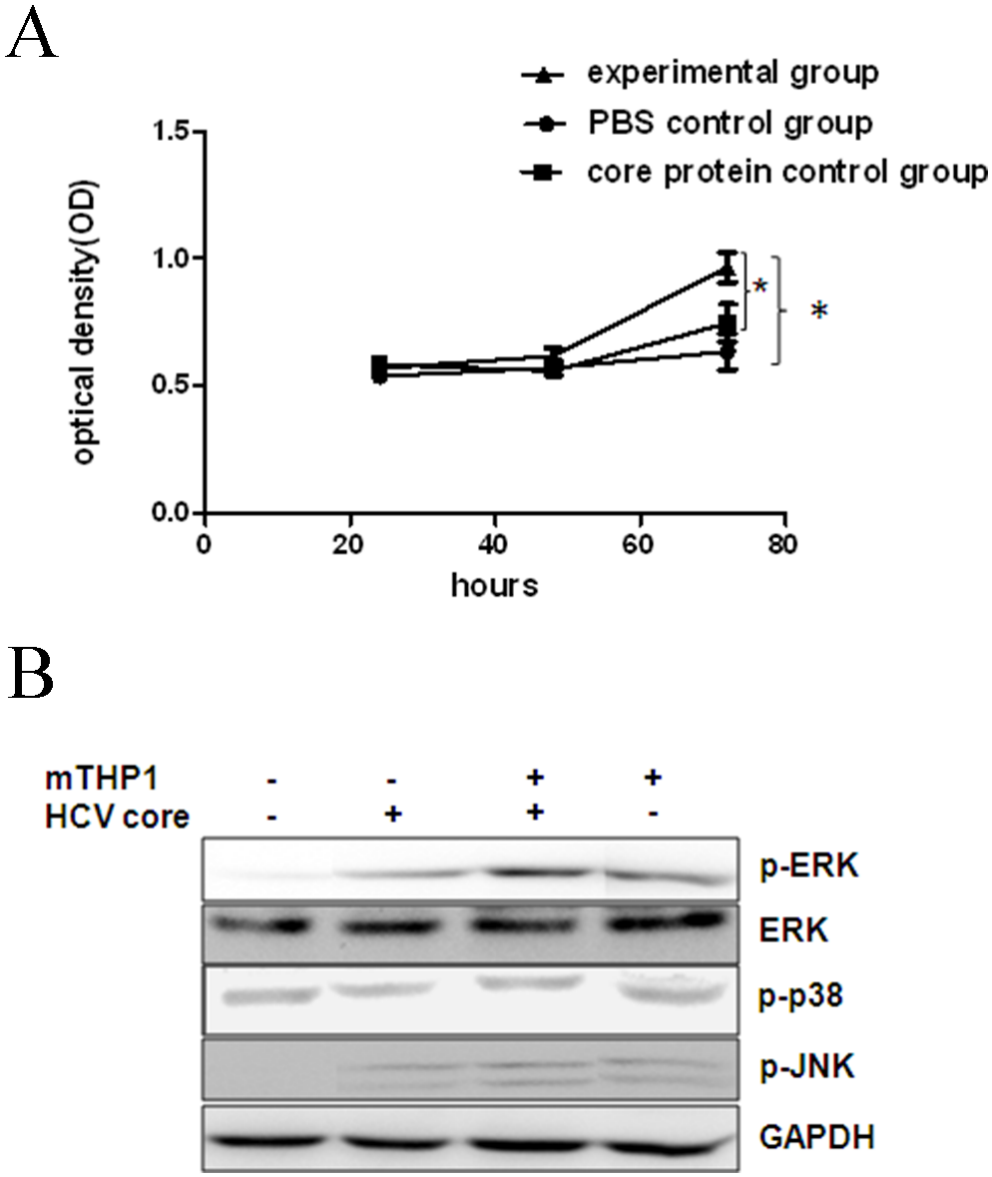
The proliferation of L02 cells. The THP-1 cells differentiated to mTHP-1 cells after incubation with PMA (40 ng/ml) for 48 h. Subsequently, Culture supernatant of mTHP-1 cells was harvested in the presence of exogenous HCV core protein (10 µg/ml) at 24 h. (A) Proliferation curve of L02 cells which were cultured in 20% of mTHP-1 cells culture supernatant and 80% of complete medium. *p<0.05 indicates alpha value as compared to control groups; (B) p-ERK protein was up-regulated in L02 cells by co-culturing mTHP-1 cells in the presence of the HCV core protein (10 µg/ml) at 24 h. The L02 cells, L02 cells only co-cultured with mTHP-1 cells, L02 cells treated with HCV core protein (10 µg/ml) respectively as control.

It was previously reported that the proliferation of hepatocytes expressing the HCV core protein was provoked [11]. However, the exogenous HCV core protein did not significantly affect the proliferation of L02 cells in this study (Figure 2A). Subsequently, L02 cells were cultured in dose-dependent concentrations of the culture supernatant from mTHP-1 cells that were treated with the exogenous core protein. The proliferation curve showed that all concentrations of mTHP-1 cells culture supernatant promoted L02 cells proliferation, and 20% cell culture supernatant showed significant proliferation of L02 cells at 72 h (Figure 2A). There was no obvious effect on L02 cells proliferation after L02 cells were co-cultured with mTHP-1 cell pellets.

ERK (extracellular signal-regulated kinase), JNK (c-junamino-N-terminal kinases) and p38 are important proteins in MAPK (mitogen-activated protein kinase) signaling pathways, which participate in the regulation of cell proliferation, differentiation, transformation and apoptosis [17], [18]. In a transwell assay system, we attempted to detect the phosphorylation of ERK, JNK and p38 in L02 cells that were co-cultured with mTHP-1 cells in the presence of exogenous HCV core protein. The expression of phosphorylated ERK was significantly increased in L02 cells, but phosphorylated JNK and p38 were unchanged (Figure 2B).

### Activity changes of mTHP-1 cells under being treated with the exogenous HCV core protein

**Figure 3:**
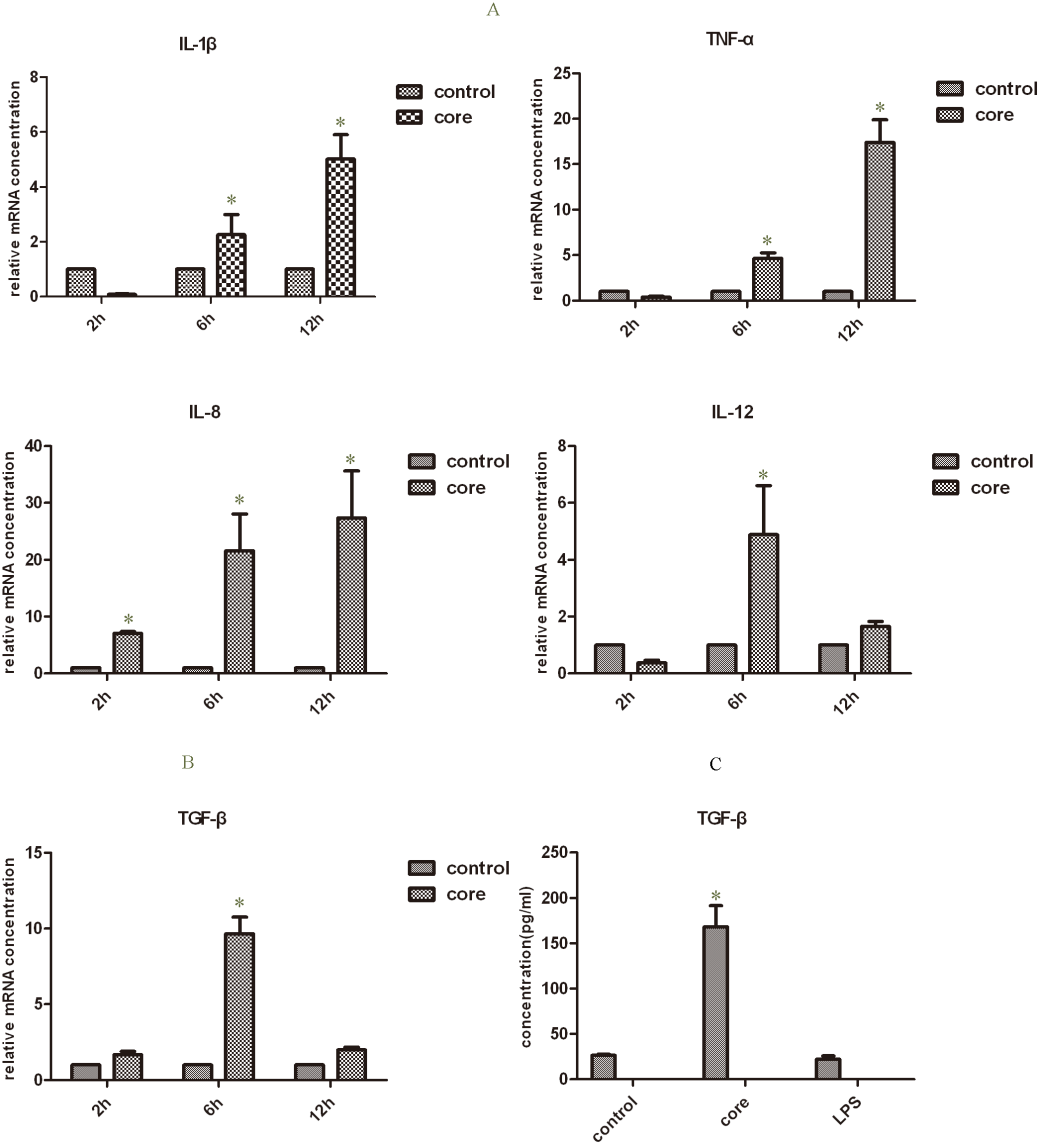
Real-time PCR and ELISA analysis of cytokines that produced by mTHP-1 cells treated with exogenous HCV core protein (10 µg/ml). (A, B) The mRNA level of pro-inflammatory and anti-inflammatory cytokines. *p<0.05 indicates alpha value as compared to PBS control group; (C) The expression level of TGF-β protein from the supernatant of mTHP-1 cells treated with exogenous HCV core protein at 24 h. *p<0.05 indicates alpha value as compared to LPS (1 µg/ml) or PBS control groups.

**Figure 4:**
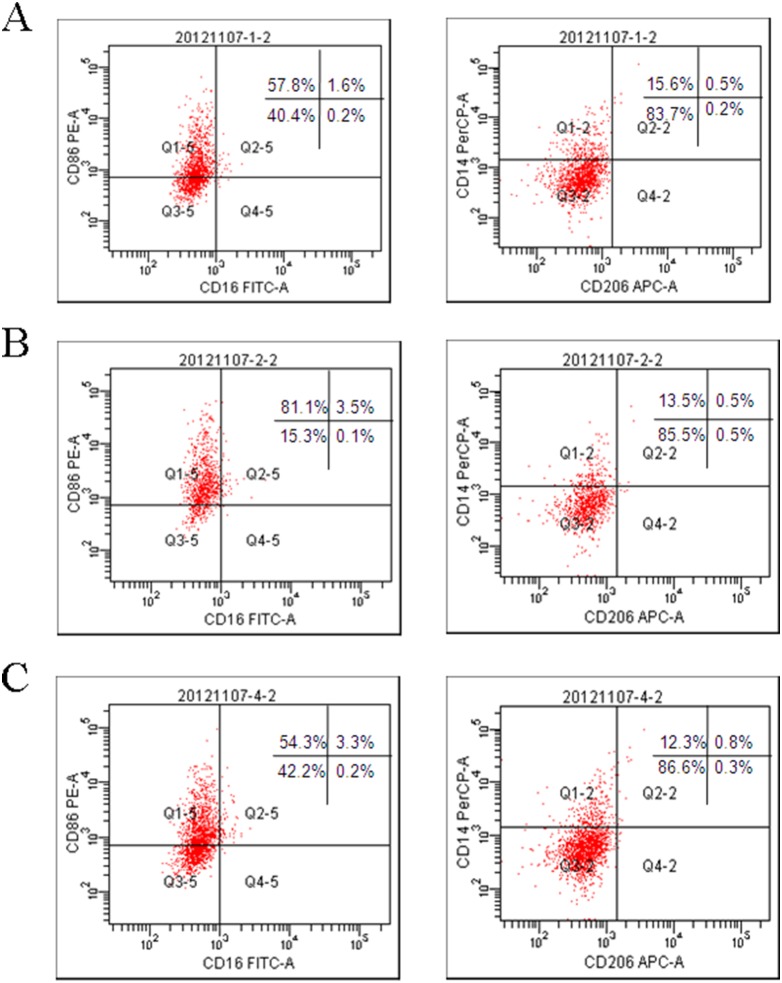
Flow cytometry plots of macrophage markers. The macrophage markers (CD14, CD16, CD206 or CD86) were analyzed in mTHP-1 cells treated with exogenous HCV core protein (10 µg/ml) for 24 h. (A) Treated with PBS as control; (B) Treated with LPS (1 µg/ml) as control; (C) Treated with HCV core protein (10 µg/ml).

**Figure 5:**
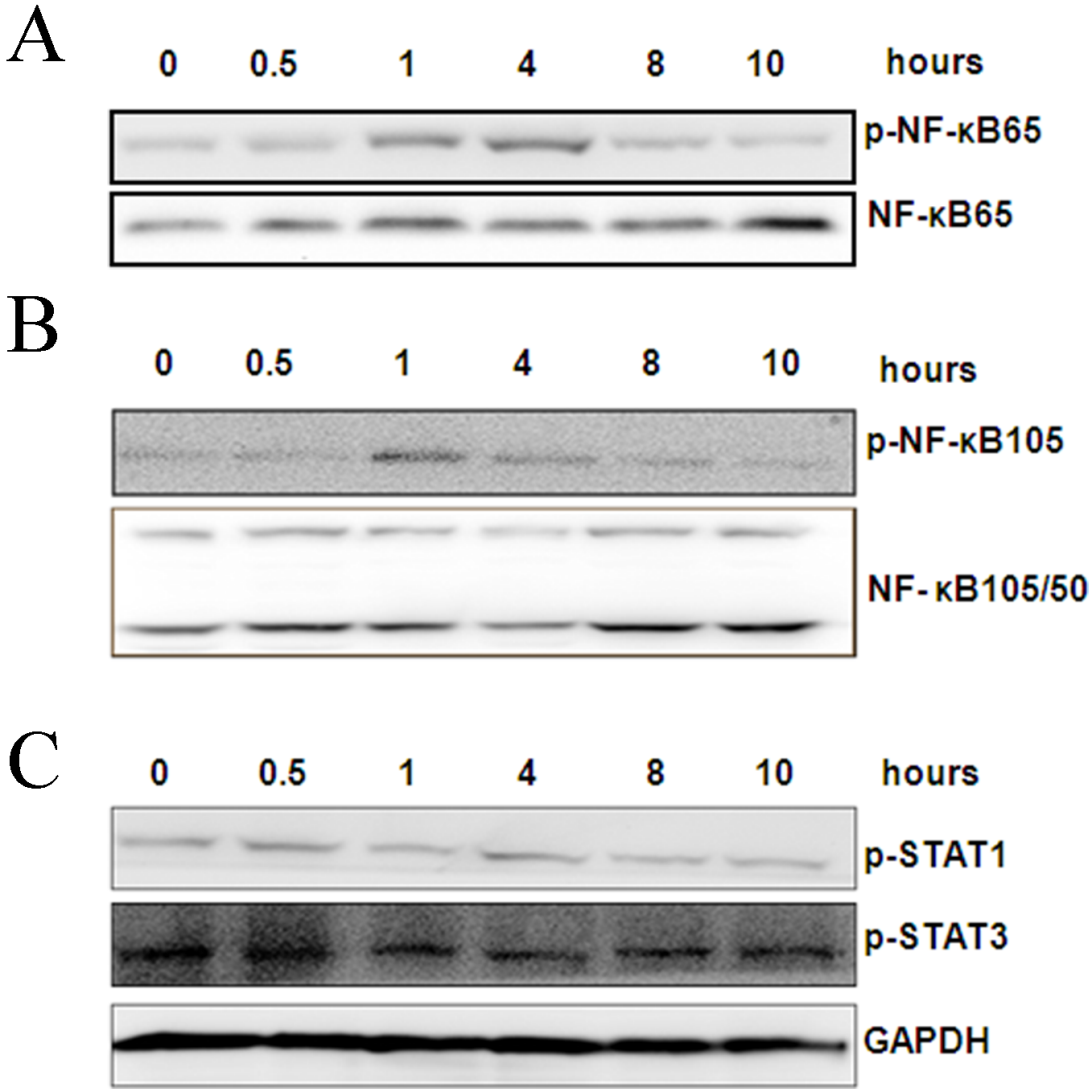
Western blot of transcription factors of mTHP-1 cells treated with exogenous HCV core protein (10 µg/ml) at different time-point (0, 0.5, 1, 4, 8, 10 h). (A) p-NF-κB65; (B) p-NF-κB105; (C) p-STAT1 and p-STAT3.

Supernatant from mTHP-1 cells that were treated with the exogenous HCV core protein increased the proliferation of L02 cells, which suggests that the HCV core protein can affect the activation of mTHP-1 cells. There were studies to provide insights into the causes of liver cell proliferation that were dependent on the changes in cytokines secretion or expression of membrane-associated molecules on mTHP-1 cells. RT- PCR showed that IL-1β, TNF-α, IL-12, IL-8, and TGF-β was significantly increased at different times (Figures 3A and 3B). ELISA analysis showed that TGF-β secretion was significantly higher at 24 h and the peak was 168 pg/ml (Figure 3C). Flow cytometry showed that the macrophage phenotype-associated surface molecules CD14, CD86, CD16, CD206 expression levels were unchanged as compared to the PBS control group. However, elevated expression of CD86 was observed in the LPS control group (Figures 4A, B, and C). Western blot analysis showed that phosphorylated NF-κB65 and NF-κB105 increased by 1 h and 4 h (Figures 5A and 5B), although there was no obvious change in the expression of phosphorylated STAT3 and STAT1 (Figure 5C).

### Phosphorylated STAT3 and membrane molecule CD206 increased in mTHP-1 co-cultured with L02 in the presence of exogenous HCV core protein

**Figure 6:**
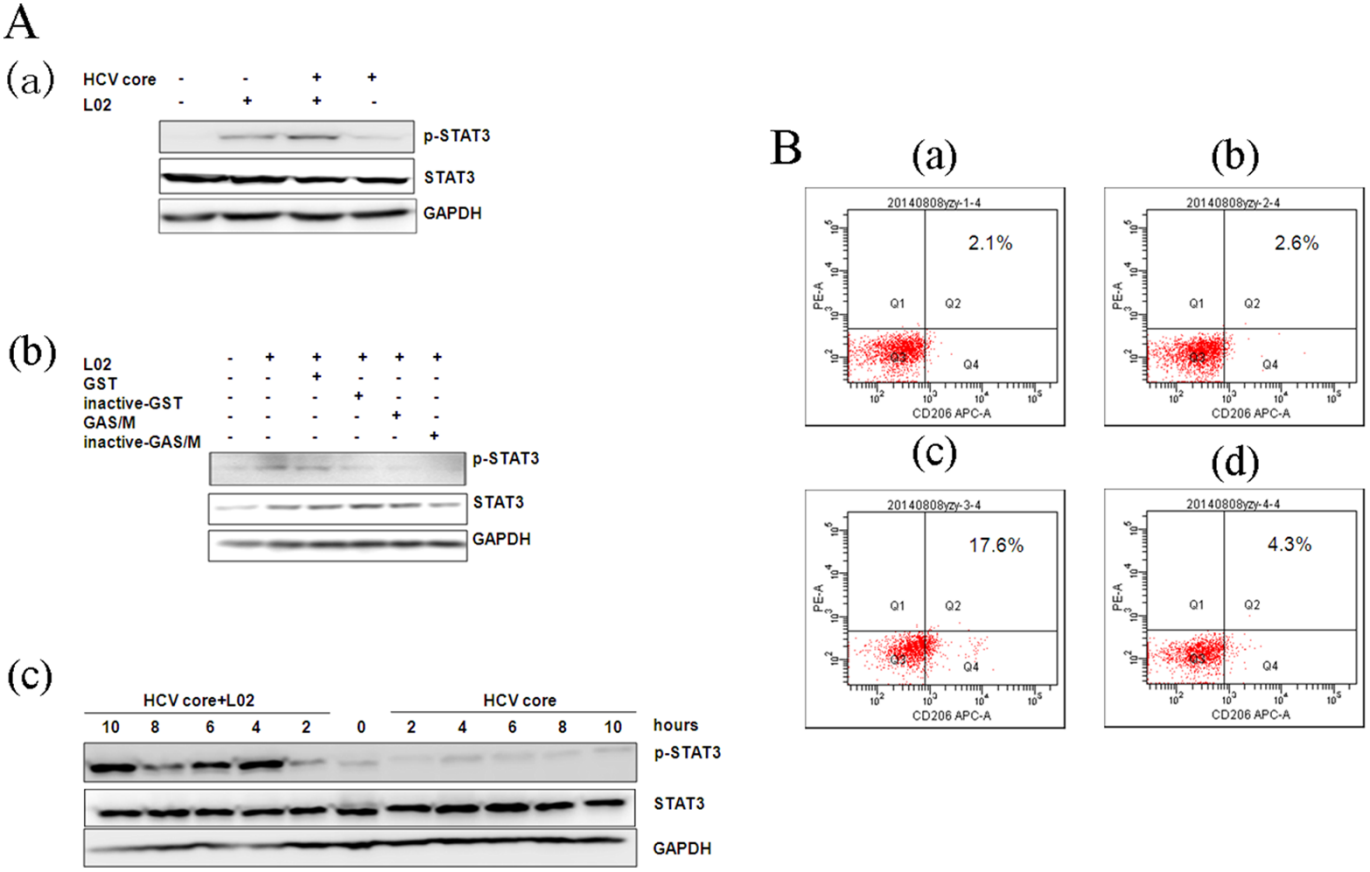
p-STAT3 and CD206 is upregulated in mTHP-1 cells. A. mTHP-1 cells co-cultured with L02 cells by transwell co-culture system in the presence of exogenous HCV core protein (10 µg/ml) or unrelated protein (GST, GAS/M protein), at indicated time-points the mTHP-1 was harvested to perform Western blot. (a) In the presence of the exogenous HCV core protein at 4 h; (b) In the presence of respective GST, GAS/M, inactive-GST, inactive-GAS/M protein at 4 h; (c) In the presence of exogenous HCV core protein (10 µg/ml) at different time-points (0, 2, 4, 6, 8, 10 h). B. mTHP-1 cells were co-cultured with L02 cells and the exogenous HCV core protein (10 µg/ml) for 24 h, then stained with APC-labeled CD206 specific antibody. The percentage of CD206-positive cells was analyzed by flow cytometry (showed in the upper-right corner). (a) mTHP-1 cells (PBS control group); (b) mTHP-1 cells were co-cultured with L02 cells; (c) mTHP-1 cells were co-cultured with L02 cells and the exogenous HCV core protein; (d) mTHP-1 cells were treated with the exogenous HCV core protein.

The HCV core protein stimulated activation of mTHP-1 cells, which subsequently promoted the proliferation of L02 cells. However, in the liver micro-environment, the HCV core protein, hepatocytes and macrophages simultaneous co-exist, so this means that the HCV core protein can mediate a role in the interaction between L02 cells and mTHP-1 cells. The transcription factor STAT3, which is a distinguishing feature of macrophage polarization to the M2 sub-population, was detected in mTHP-1 cells when they were co-cultured with L02 cells in the presence of exogenous HCV core protein or the unrelated protein. These observations informed us that phosphorylated STAT3 expression in mTHP-1 cells in the presence of the HCV core protein was significantly increased at 4 h (Figures 6A–a). However, phosphorylated STAT3 was not obviously changed in the presence of the unrelated protein (e.g., GST protein, inactive-GST protein, GAS/M protein, or inactive-GAS/M protein) (Figure 6A–b). And follow-up experiments confirmed that phosphorylated STAT3 was stimulated by 4 h to 10 h in the experimental group (Figure 6A–c). Subsequently, to directly assess if L02 cells and the exogenous HCV core protein induce an M2 phenotype in macrophages, we determined the expression of mannose receptors (CD206), a well-accepted marker for M2 macrophages [19], in mTHP1 cells by using flow cytometry. The results showed that the the percentage of CD206-positive macrophages was increased in the experimental group (Figure 6B).

**Figure 7:**
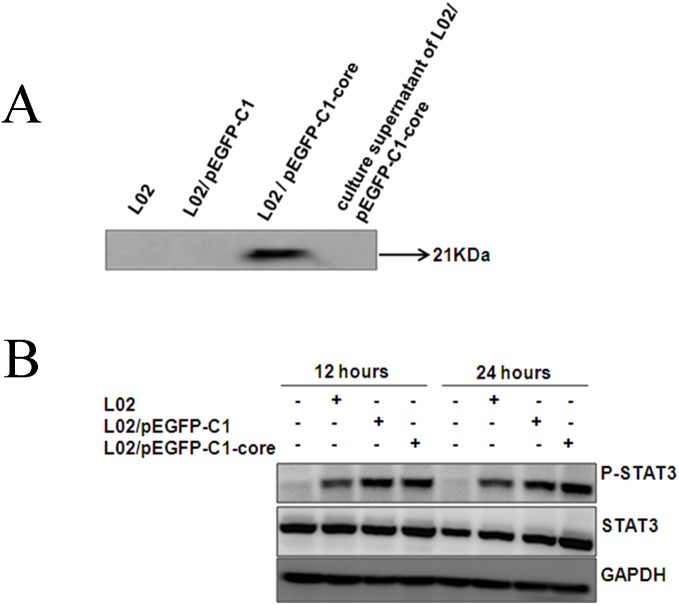
The endogenous HCV core protein effect on p-STAT3 expression. The pEGFP-C1/HCV core plasmid transfected L02 cells were co-cultured with mTHP-1 cells. (A) Western blot analysis of the HCV core expressed in L02 cells; (B) Western blot analysis of p-STAT3 in mTHP-1 cells which co-cultured with HCV core gene transfected L02 cells at indicated time-points (12 h and 24 h).

The above studies inferred that the increased phosphorylation of STAT3 in mTHP-1 cells was related to the exogenous HCV core protein. Thus, we obtained L02 cells that were transfected with the pEGFP-C1/HCV core plasmid. Subsequently, the HCV core protein was located within L02 cells instead of in the secreted form as detected in the culture supernatant (Figure 7A). We found it surprising that phosphorylated STAT3 was not obviously changed in mTHP-1 cells when they were co-cultured with L02 cells that had been transfected with core-gene (Figure 7B).

## Discussion

Increasingly, experimental data suggests that persistent HCV infection is the main mechanism of HCC [1], [2]. HCV is a hepatotropic and single strand RNA virus that is composed of 9400 nucleotides coding four structural proteins (e.g., core, p7, E1 and E2) and six nonstructural proteins (e.g., NS2, NS3 and NS5B) [20]. As an important structural protein, HCV core protein is an important part of the viral nuclear capsid [21], which affects the proliferation, differentiation and apoptosis of hepatocytes through a variety of mechanisms [9], [10], [22]. In recent years, many studies have explored the mechanism of HCV core protein influencing the functions of hepatocytes. For example, the core protein can promote 5-FU-mediated p53-dependent caspase-7 activation to inhibit hepatocyte HepG2 growth [23], or promote HepG2 proliferation and anti-apoptosis through up-regulating autocrine secretion of HB-EGF (heparin-binding EGF-like growth factor) and activating Akt by the Ras/PI(3)K pathway [11]. Additionally, the core protein mediates the activation of stellate cells, which is dependent on TGF-β secretion by Huh7 cells [12]. Due to the lack of an effective cell culture system of HCV *in vitro*, the various research findings closely depend on the selection of target cells or the HCV core protein that might be derived from different HCV subtypes. Experimental and clinical data have showed that HCV core protein is not only located in the cytoplasm and nucleus, but is also secreted by stably transfected cells and infected cells. And this free core protein has been shown to affect the biological activities of target cells through its interaction with a complement receptor, C1qR found on the surface of immune cells, such as T lymphocytes, macrophages, and dendritic cells [24]–[28]. It suggests that the HCV core protein can be used as a surrogate ligand to modulate other cellular activities and functions in the liver micro-environment besides affecting hepatocytes by as yet, unidentified mechanisms.

Kupffer cells (KCs), resident liver macrophages, are long lived and abundant, representing 15 to 20% of the total liver cell population, and play an important role in immune surveillance and immune regulation in the early stages of microbial infection [29]–[31]. However, the mechanism and function of KCs in persistent infection with HCV remains unclear. Studies have reported that HCV core protein combined with C1qR of macrophages inhibits activation of GSK-3β and ASK1, which damages the intracellular MAPK pathway and then inhibits the production of IL-12 of macrophages [32], [33]. It is well-known that macrophages respond to diverse environmental signals by expressing any one of an array of functional phenotypes [34]. Despite this diversity, two distinct macrophage activation states have been recognized: the M1 (or classically activated) macrophage sub-type and the M2 (or alternatively activated) macrophage sub-type. The M1 macrophages is characterized by increased microbicidal activity (including the expression of NOS2), high antigen-presenting activity associated with increased MHC class II expression, and expression of the co-stimulatory molecules (CD80 and CD86), and increased production of pro-inflammatory factors (e.g., IL-1β, IL-12, and TNF-α). It is clear that STAT1 activity is crucial for M1 macrophage polarization. The M2 macrophages are thought to be involved in dampening inflammation, and produce high levels of anti-inflammatory cytokines (e.g., IL-10 and TGF-β), display low antigen-presenting ability, and activation of STAT3 might be an important role in M2 macrophage polarization [35], [36]. The M2 macrophages are distinguished from other macrophage populations by several markers including the IL-4R, mannose receptor (MR/CD206), Arg1 and Ym1/2 (eosinophilic protein from chitinase family) and so on [19]. The imbalance between M1 and M2 often lead to pathological disease, including an increase in M1 macrophages that can lead to chronic inflammatory diseases, and an increase in M2 macrophages that can promote severe immunosuppression. Interestingly, M1 and M2 phenotypes might not be stably differentiated subsets. For example, LPS-activated macrophages become unable to reactivate a large fraction of their pro-inflammatory genes a few hours following re-stimulation, and yet they retain the ability to induce the expression of anti-inflammatory cytokines (e.g., IL-10) *in vitro* [37]; in early tumor development, local macrophages are the M1 phenotype, while after tumor formation, local macrophages differentiate to the M2 phenotype [38]. Indeed, there is plasticity in macrophage programming, which causes macrophages to switch readily from one functional phenotype to another, or share the different phenotype of macrophage sub-populations at certain stages in response to new microenvironmental signals [19].

In the settings of persistent infection by HCV, the liver micro-environment is altered concordantly with the evolution of disease, which may cause some changes in hepatocyte functions and macrophage activities. Subsequently, hepatocytes and macrophages interact in a way by which we believe that the virus or viral proteins can mediate the interaction between hepatocytes and macrophages. However, prior studies have not been reported to demonstrate this phenomenon. At the same time, despite clinical and animal model studies that have explored the correlation between the different cytokines in the liver micro-environment and persistent infection with HCV [39], [40], that the specific cell types in the liver that were induced to secrete various cytokines during the evolution of disease is still unclear.

Thus, in this study, we discussed the interaction among the HCV core protein, hepatocytes and macrophages in multiple ways.

The results showed that the culture supernatant from mTHP-1 cells treated with the exogenous HCV core protein induces significant L02 cells proliferation, while the co-culture of mTHP-1 and L02 cells (a culture system of direct cell contact) or the HCV core protein failed to induce marked proliferation of L02 cells. The results also confirmed that phosphorylated ERK was increased in L02 cells that were co-cultured with mTHP-1 cells and in the presence of the HCV core protein. It suggests that the exogenous HCV core protein mediates a role in the interaction between human hepatocytes proliferation and macrophage activities.

Thus, subsequent studies set out to observe the direct effects of the exogenous HCV core protein on the activities of the macrophages (mTHP-1), including the changes in the expression of cytokines, membrane-associated molecules, and signal transduction molecules. The results showed that the core protein induced mTHP-1 cells to secrete predominantly cytokines including anti-inflammatory cytokines like TGF-β, and pro-inflammatory cytokines like IL-8 and TNF-α. Moreover, TGF–β can also be detected in cell culture supernatant. TGF–β is a cytokine with multifunctional properties that are involved in the regulation of the immune response, cell cycle, differentiation, and apoptosis [41]. Benzoubir [12] reported that TGF–β is detected in the cell culture supernatant of hepatocytes that express the core protein rather than any other non-structural protein, which leads to phosphorylation of Smad2 by an autocrine mechanism, then in turn activates stellate cells. And IL-8 also was secreted by hepatocytes that expressed the core protein, and that this might contribute to the establishment of a profibrogenic micro-environment by inducing smooth muscle actin expression in hepatic stellate cells [42]. Thus, our studies also suggest that the expression of TGF–β or IL-8 by macrophages in the presence of exogenous HCV core protein contributes to chronic HCV infection. Of note, both CD14 and CD86 (which are important co-stimulatory molecules in antigen presentation) are associated with the phenotype of M1 macrophages, and both CD16 and CD206 are associated with the phenotype of M2 macrophages [43]. However, the expression of membrane-associated molecules (e.g., CD14, CD16, CD206, and CD86) by mTHP-1 cells treated with exogenous HCV core protein was not significantly different as compared with the PBS control group. These results were also consistent with the changes seen in L02 cell proliferation, which was caused by the supernatant from mTHP-1 cells rather than by direct cell-to-cell contact. However, there was evidence of elevated levels of phosphorylated NF-κB65 and NF-κB105, which was accompanied by the secretion of cytokines, whereas phosphorylated STAT3 and STAT1 remained almost unchanged. These results suggest the HCV core protein did not induce macrophages to differentiate through a classical pathway of activation, but provoked macrophage activation to an intermediate state, sharing functional phenotype of both M1 and M2 macrophages. However, due to the changes in the liver micro-environment during the progression of disease and the flexibility of macrophage programming, the choice of experimental time is very important in the context of further studies.

Surprisingly, phosphorylated STAT3 in mTHP-1 cells that were co-cultured with L02 cells in the presence of the exogenous HCV core protein was significantly higher, as compared to mTHP-1 cells that were co-cultured with L02 cells in the presence of an unrelated protein, or with L02 cells that expressed the core protein in cells. Consistent with the above results, the expression of CD206 in the experimental group was increased. It is hypothesized that the exogenous core protein stimulates hepatocytes via specific pathway to induce a distinct set of molecules, and this culminates in the generation of altered macrophage subtypes in the local liver micro-environment, however which cell signaling response to macroghages in the interaction among macroghages, hepatocytes, and the exogenous HCV core protein will be proved in the future experiment.

In summary, the exogenous HCV core protein mediates an important role in the interaction between human hepatocyte proliferation and macrophage subtypes, which may involve in persistent infection by HCV. However, when considering the diversity of HCV proteins and the complexity of the *in vivo* environment, many events, including the effects of other HCV proteins on macrophages, the interaction between macrophages and other cells (e.g., immune cells, primary hepatocytes, and stellate cells), and other intracellular downstream molecules, are all involved in the process of persistent HCV infection. Therefore, additional studies are warranted to explore the specific molecular mechanisms that are involved in the differentiation of macrophages, then systematically analyze the interaction among cells, molecules, and proteins in the process of persistent HCV infection. These data provide a theoretical basis and the methods for the potential clinical treatment of patients presenting with chronic HCV infection.
